# “Re-Think” Sulfur Curing

**DOI:** 10.3390/molecules29215198

**Published:** 2024-11-02

**Authors:** Anke Blume, Frances van Elburg, Fabian Grunert, Auke Talma

**Affiliations:** Chair of Elastomer Technology & Engineering, Department of Mechanics of Solids, Surfaces & Systems (MS3), Faculty of Engineering Technology, University of Twente, 7522 NB Enschede, The Netherlands; f.a.vanelburg@utwente.nl (F.v.E.); f.grunert@utwente.nl (F.G.); a.g.talma-1@utwente.nl (A.T.)

**Keywords:** sulfur curing, SSBR, vinyl content, accelerator, Zinc oxide, stearic acid

## Abstract

Since Charles Goodyear discovered the method of sulfur curing Natural Rubber in 1839, many studies have been carried out to understand its mechanism. Currently, the broadly accepted mechanism includes an activated accelerator complex formed by Zinc oxide, stearic acid, accelerators and sulfur. Furthermore, it is also broadly accepted that the coupling of the sulfur to the polymer takes place in the allylic position to the double bond. Modern passenger car tire treads no longer contain Natural Rubber but instead a blend of Solution Styrene Butadiene Rubber and Butadiene Rubber, filled with a silica/silane system. Is it possible to transfer all the gained knowledge from the Natural Rubber crosslink reaction to such modern passenger car tire tread formulations, or is it required to “re-think” sulfur curing?

## 1. Introduction

**Figure 1:**
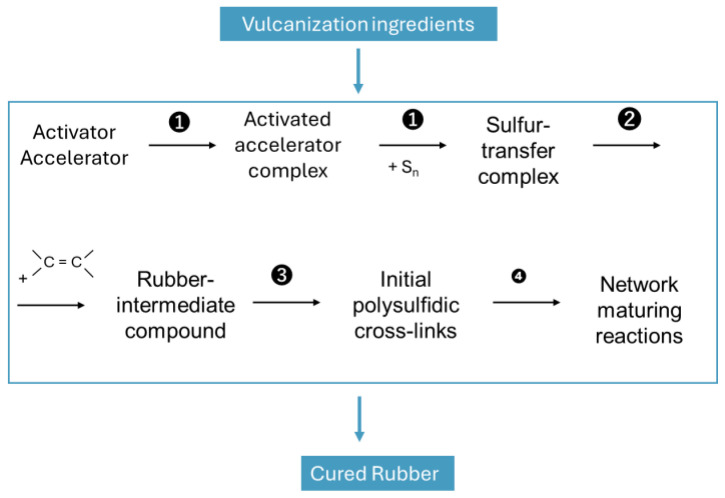
Sulfur curing mechanism by substitution reaction at the allylic position according to [3,4].

Since Charles Goodyear discovered the sulfur curing of Natural Rubber (NR) in 1839 [1,2], many studies have been carried out to understand its mechanism. The broadly accepted mechanism was summarized by Morrison and Porter (Figure 1) [3].

**Figure 2:**
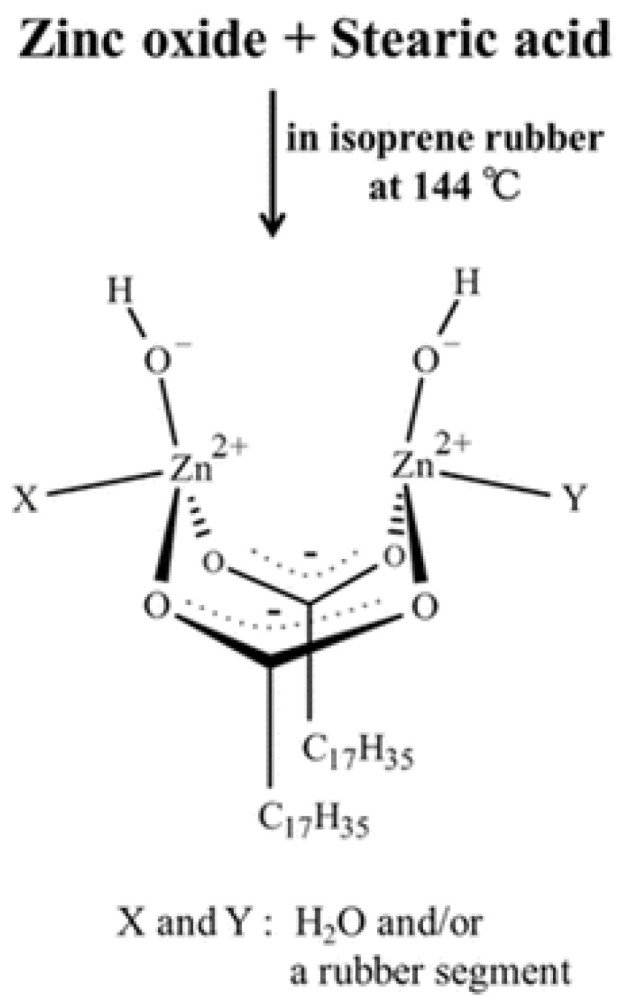
Dinuclear bridging bidentate Zinc/stearate complex according to [5].

In general, sulfur attaches at the allylic position to the polymer. It is activated by the inclusion of an activated accelerator complex formed by Zinc oxide and stearic acid where accelerator fragments are attached as ligands. Ikeda [5,6] investigated this intermediate complex further and concluded that it is a dinuclear bridging bidentate Zinc/stearate complex with the following structure: (Zn_2_(μ-O_2_CC_17_H_35_)_2_)^2+^(OH^−^)_2_·XY, where X and Y are water and/or a rubber segment (Figure 2). No information is given how the hydrogen atom is abstracted by this complex to enable the final polymer–polymer coupling.

**Figure 3:**
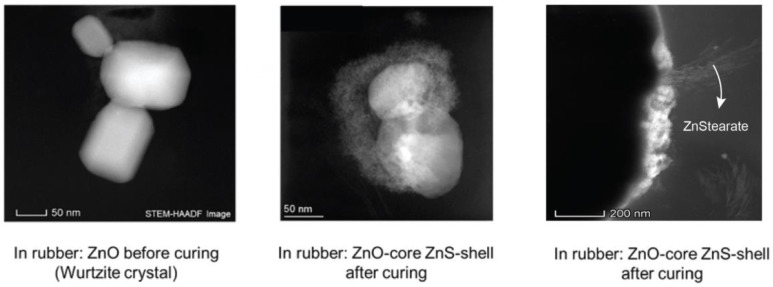
Chemical reactions take place at the surface of the Zinc oxide (ZnO) crystal in the rubber matrix [7].

The place where the complex is formed was further investigated by Butuc. She found out that its formation takes place only at the surface of the Zinc oxide (ZnO) crystal. At the end of the curing reaction where sulfur bridges between the polymer chains are formed, Zinc Sulfide (ZnS) as well as Zn(stearate)_2_ are additionally formed as side products (Figure 3) [7]. The sulfur crosslink formation is not homogeneous but localized, resulting in highly crosslinked (ZnS- and Zn-stearate rich) as well as un-crosslinked (ZnS- and Zn-stearate deprived) regions [8].

**Figure 4:**
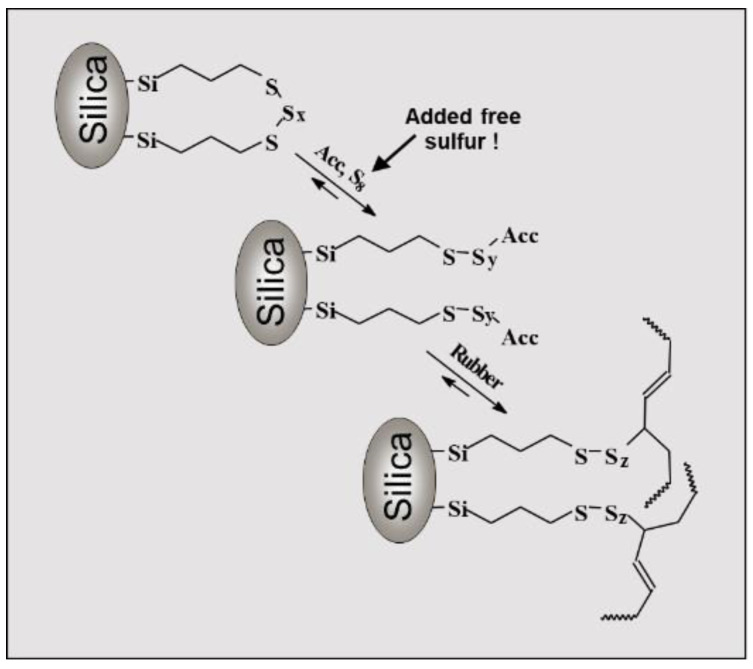
Silica/silane/polymer coupling via the sulfur function of the silane to the allylic position of the polymer [10].

These more general findings can be used to understand the reactions during the production of a tire tread compound in a better way. A modern green tire tread compound is based on a silica/silane-filled Solution Styrene Butadiene Rubber (SSBR)/Butadiene Rubber (BR) compound [9]. In addition to the coupling of the polymer chains via sulfur bridges, a new focus has to be put on the silica/silane/polymer bonding via the sulfur function of the silane in such a compound. It was assumed that the sulfur function of the silane, typically a bis(triethoxysilylpropyl)tetrasulfide (TESPT), is also connected to accelerators, which are part of the active complex (Figure 4) [10].

Connecting the assumption of the coupling of the silane to the active complex to the latest findings of Ikeda and Butuc would lead to the conclusion that the active Zinc complex does not contribute to the formation of the polymer–filler bonds in the assumed way. If the complex is bound to the Zinc oxide surface where it supports the coupling of the sulfur to the mobile polymer chains, it is unlikely to assume that the same supporting function takes place for the silane coupled to the non-mobile silica particle. Zinc oxide as well as silica particles are distributed in the polymer matrix; such a supporting function of the complex would have the pre-condition that both solid particles are in connection to each other, which is usually not the case [11].

**Figure 5:**
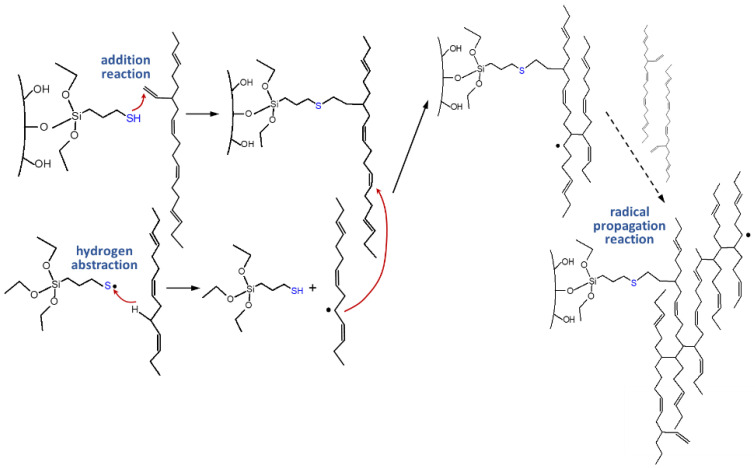
Coupling mechanism of mercaptosilanes in a green tire tread compound according to [12].

This polymer–filler crosslink reaction between silica and polymer via silane was further investigated by introducing an alternative group of sulfur silanes, the mercaptosilanes. The use of these mercaptosilanes as an alternative to the traditional tetra or disulfide silanes led to an improved dispersion behavior of the silica, an improved rolling resistance, but also to a deteriorated processing behavior. Sato investigated their behavior in depth and concluded that the observed effects were based on a combination of an addition reaction and a radical propagation reaction, where the sulfur radical of the mercaptosilane couples directly to the vinyl group of the main polymer, the SSBR, following an Anti-Markovnikov mechanism. This radical reaction leads to the quick generation of a large amount of dense chemical bound rubber, resulting in the observed good dispersion which causes the very low rolling resistance. Due to the fact that this coupling to the vinyl group already takes place during the mixing, the processing was worse and the viscosities increased (Figure 5) [12].

**Figure 6:**
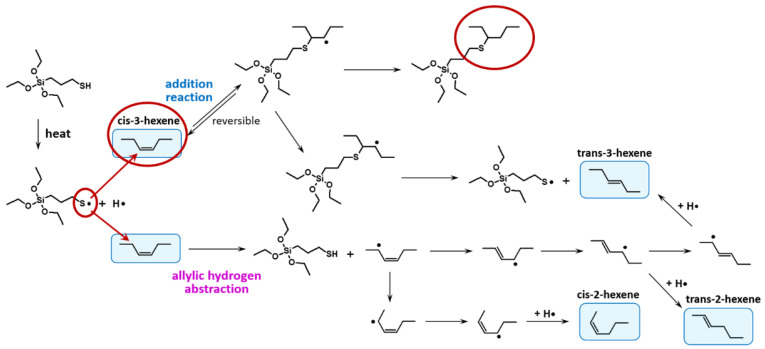
Addition reaction of a sulfur radical of a mercaptosilane to a cis double bond according to [12].

Furthermore, he investigated the possibility of also coupling to cis double bonds and confirmed, using model studies with alkenes as well as oligomers, that a direct coupling from the sulfur radical of the mercaptosilane to the cis double bond can occur, only with a lower yield than the coupling to the vinyl group (Figure 6). Additionally, cis/trans isomerization could be observed [12].

In summary, it can be concluded that a sulfur radical of a mercaptosilane can couple directly to a vinyl double bond as well as to a cis double bond, although at a lower yield. This means that the previous assumed support of the Zinc complex for a faster coupling reaction is not required in this system. This leads to the consideration that a similar process might also explain the coupling of the silane-modified silica to the polymer. If this coupling indeed follows a different mechanism as assumed, it raises the question if the whole sulfur coupling in an SSBR/BR-based tire tread compound has to be re-considered.

## 2. Research Questions

The following main research questions should be answered in the current study: If a sulfur radical from a mercaptosilane can couple directly, preferably to a vinyl double bond, can such a direct coupling also occur from an opened sulfur ring to the vinyl group? Is this opening triggered only by temperature or is the presence of supporting molecules like accelerators required? Is it required to “re-think” the traditional coupling of the sulfur to the allylic position of the polymer, as described before?

## 3. Re-Evaluation of Literature Data

**Figure 7:**
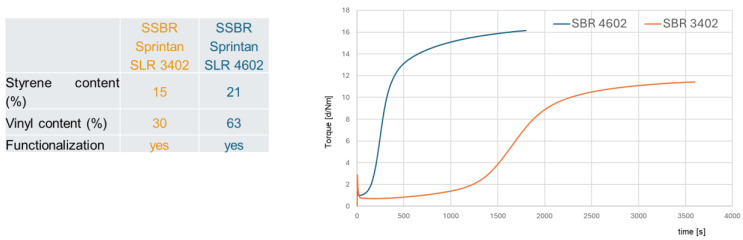
Curing behavior of a Solution Styrene Butadiene Rubber (SSBR) with low/high-vinyl content according to [13].

To answer these questions, firstly, the curing behavior of sulfur-cured high-vinyl and low-vinyl SSBR was investigated. Such a study is already described in the literature (Figure 7) [13]. The curing behavior of high- and low-vinyl SSBR was investigated in a model green tire tread compound with 2.5 phr *N*,*N*’-Diphenylguanidine (DPG), 0.2 phr Tetrabenzylthiuram disulfide (TBzTD), 1.6 phr *N*-cyclohexylbenzothiazole-2-sulphenamide (CBS) and 2 phr sulfur.

It can be observed that the presence of a high-vinyl content leads to much faster curing and a higher final torque. The higher vinyl SSBR (Sprintan SLR 4602) also has a higher amount of styrene. This means that it has less butadiene units which results in less allylic positions to couple to. Therefore, a lower torque should be expected when the torque is directly connected to the crosslink density without changing any other parameters like filler dispersion. Furthermore, the cure rate should be the same if the same coupling mechanism is assumed. This aspect was not yet considered in this study.

## 4. Proposal for an Alternative Sulfur Coupling Mechanism

**Figure 8:**
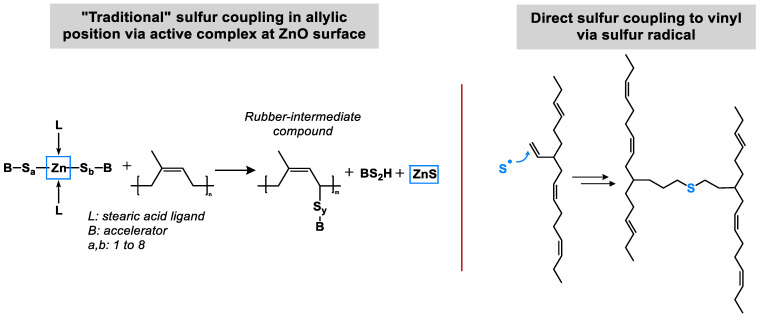
Two different mechanisms of sulfur coupling.

This is the first hint which can be found in the literature when the already presented results are re-evaluated with a new focus. It can be assumed that two different mechanisms have to be considered for sulfur coupling. The “traditional” sulfur coupling occurs mainly in the allylic position, often via an active complex at the ZnO surface. The alternative, a direct sulfur coupling, would occur by a direct binding of a sulfur radical to a vinyl double bond (Figure 8). This direct coupling needs a trigger for the opening of the sulfur ring to form the sulfur radical. It can be the impact of the temperature or the accelerator or a combination of both. It is assumed that the Zinc complex does not play a role here.

The big difference (besides the position where the sulfur couples) between both mechanisms is that the “traditional” sulfur coupling needs activators but the direct coupling does not. Therefore, the logical approach to investigate this further is to cure high- and low-vinyl SSBR with and without activators.

## 5. Curing Behavior of High-Vinyl SSBR Versus Low-Vinyl SSBR

To answer the question if high-vinyl SSBR follows a direct radical coupling mechanism and does not need any activators for this, two different SSBR types were investigated: the SSBR Sprintan SLR 3402 with a low and the SSBR Sprintan SLR 4601 with a high-vinyl content (Table 1).

**Figure 9:**
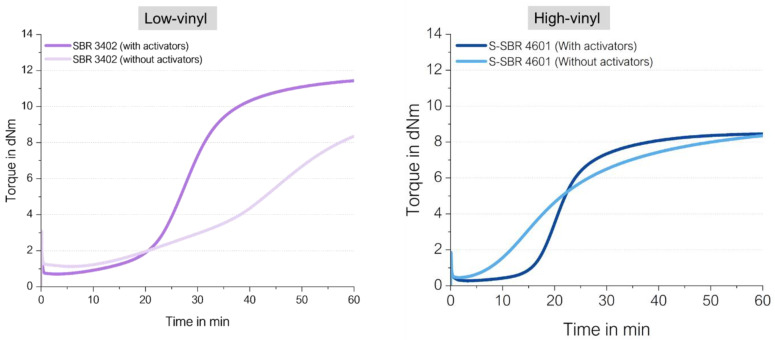
Curing behaviors of a low-vinyl (**left**) and a high-vinyl (**right**) unfilled SSBR compound with and without activators.

Each of these two types were tested in a very basic unfilled compound with 2.0 phr *N*-tert-butyl-2-benzothiazyl sulphenamide (TBBS) and 1.4 phr sulfur and compared to the same compounds, where additional 2.5 phr ZnO and 2.5 phr stearic acid (called in the following “activators”) were added. Figure 9 shows their different curing behaviors. Additional information about the materials and methods used in this chapter are displayed in Appendix A.

There are several remarkable observations, starting from the graph on the right-hand side: the high-vinyl SSBR shows the same final torque with and without activators. This already delivers the first hint that the above proposed direct radical mechanism from an opened S8 ring to a vinyl bond indeed takes place. Chapman and Johnson already considered this direct coupling as well; they proposed that “Zinc-accelerator complexes are either not needed or are acting catalytically” in an SSBR [14].

Furthermore, the curing of high-vinyl SSBR starts earlier without activators and the scorch time is significantly lower. This behavior can also be explained by the assumed radical process: a direct sulfur coupling to vinyl via sulfur radical starts earlier than the “traditional” ionic process but has a lower reaction rate. Normally, radical processes can be assumed to be very fast and can even cause explosions [15]. This assumes a chain reaction. In the current case of the direct coupling between a sulfur radical and the vinyl group, many different sulfur radicals need to be formed all over the matrix. The radical reaction can be terminated in many different ways; no chain reaction is assumed to take place all over the compound. This also means that the supporting substance for this formation cannot be a catalyst but a substance which is well distributed in the compound, similar to sulfur, to initiate the radical formation spread all over the compound. The other option for such a trigger is the temperature.

The presence of activators in the high-vinyl SSBR compound increases the scorch time. This leads to the conclusion that ZnO and stearic acid do not act as activators in the system but as retarders. They seem to interact with sulfur as well as with accelerators, capturing the sulfur radicals or avoiding even the formation of the radicals.

On the left-hand side, the low-vinyl-containing SSBR cures were much slower in the absence of activators, reaching only 60% of the final torque of the compound after 60 min in the presence of activators. Here, the amount of vinyl groups is too low to enable an efficient direct coupling.

The absolute value of the final torque of the low-vinyl SSBR with activators is higher in comparison to the high-vinyl SSBR. This is connected to the fact that the low-vinyl SSBR also contains a significant lower amount of styrene, leading to a higher degree of butadiene units in the SSBR and ultimately meaning a higher degree of crosslinkable sites. This higher degree of double bonds in the main chain seems to lead to a higher crosslink density indicated by the higher observed end torque.

## 6. Curing Behavior of NR

The above described surprising finding led directly to the next question: Why was this not yet reported in the literature?

The sulfur curing mechanism described in the introduction (Figure 1) was investigated for NR. NR does not contain any vinyl groups; therefore, it is considered that the direct radical coupling is not possible, which leads to the assumption that curing without activators should not lead to a sufficient torque increase.

**Figure 10:**
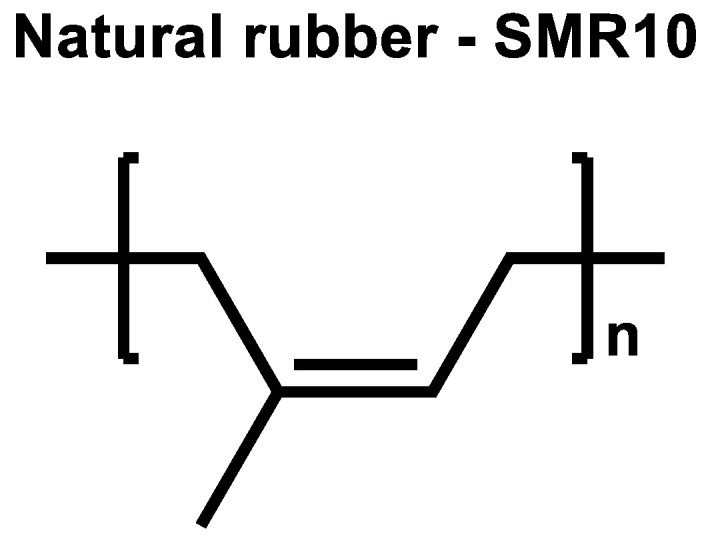
Structure of the used Natural Rubber (NR) type.

**Figure 11:**
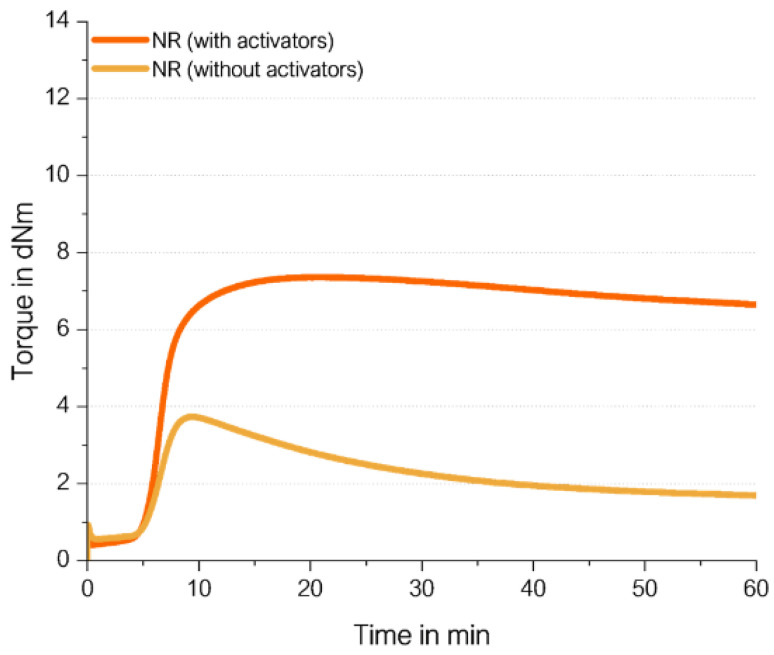
Curing behavior of an unfilled NR SMR10 compound.

To prove this, SMR10 was selected as the NR of choice (Figure 10). Its curing behavior was investigated in an unfilled compound with 2.0 phr TBBS and 1.4 phr sulfur, and was compared to the same unfilled compound where additionally 2.5 phr ZnO and 2.5 phr stearic acid were added (Figure 11). Appendix A describes the used materials and methods in detail.

The curing of NR without activators results in a very low final torque. The torque increases but only by a low amount. This proves the theory that the curing in NR follows the ionic mechanism which was already described in the literature due to the absence of vinyl groups in NR. This indicates that the presence of ZnO and stearic acid is essential to abstract the allylic hydrogen atom from the polymer chain to enable the coupling of sulfur.

The curing curve of the NR compound without activators has a maximum. The shape of the curve is similar to the curve with activators, but with a more pronounced maximum on a much lower level. The fact that NR-based compounds show a maximum caused by reversion is known from the literature [16,17]. It can be caused by the breakage of the long polymer chains of NR, as well as the polysulfidic bonds created during the vulcanization process. In the case of the curing curve of the NR compound without activators, the formation of crosslinks does occur in the presence of TBBS as an accelerator and sulfur [18]. However, the polysulfidic bonds that are created are weak and break relatively fast, resulting in strong reversion. Based on the results in Figure 11, it can be concluded that the activators are needed to create a stronger crosslinking network in the used NR matrix.

## 7. Curing Behavior of High-Vinyl BR Versus Low-Vinyl BR

If the assumed direct sulfur coupling occurs in high-vinyl SSBR, it can be expected that the same mechanism will also occur in BR. To prove this, a low-vinyl (CB24) as well a high-vinyl BR (Europrene HV80) were selected. Their cure behavior was tested in an unfilled compound with 2.0 phr TBBS and 1.4 phr sulfur with and without the addition of 2.5 phr ZnO and 2.5 phr stearic acid..

**Figure 12:**
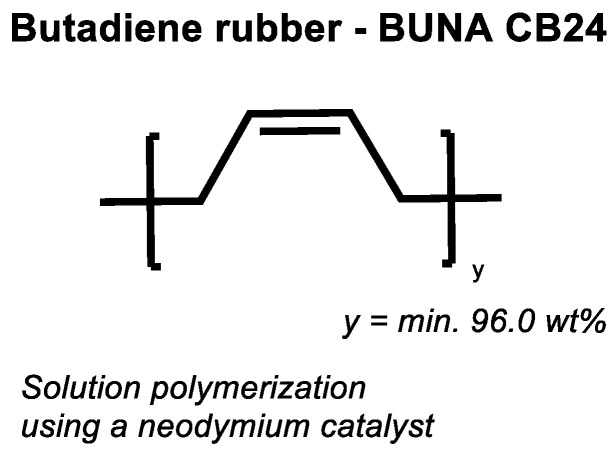
Structure of the used high-cis Butadiene Rubber (BR) type (according to [19]).

**Figure 13:**
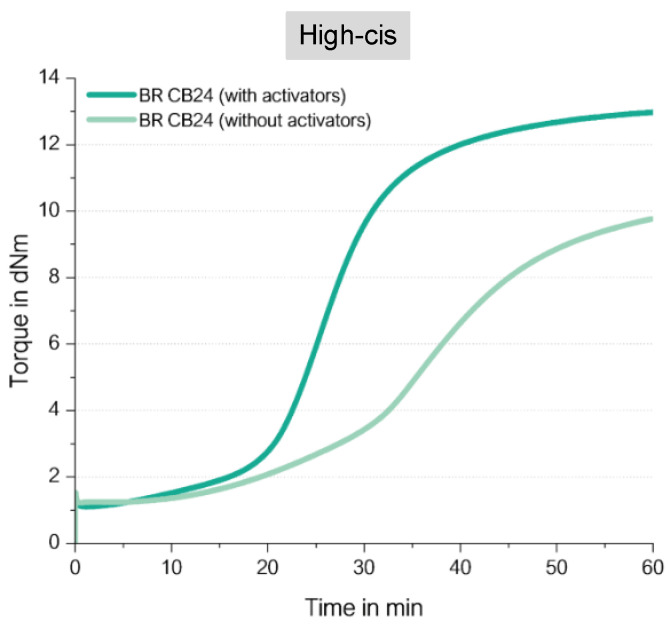
Curing behavior of unfilled low-vinyl/high cis-BR compounds.

Figure 12 shows the chemical structure of the used high-cis BR, and Figure 13 displays the curing behavior of the low-vinyl BR with 96 wt% of cis double bonds. Additional information about the used materials and methods are described in Appendix A.

It shows that the compound without any activator shows a significant increase in torque but results in a still lower final torque in comparison with the compound with activators. CB24 does contain 96 wt% of cis double bonds; therefore, this behavior was unexpected.

An answer for this can be found in the literature. M. Sato [12] reported that an alkene (cis-3-hexene) can react directly with mercaptosilane in a radical reaction and forms a variety of different reaction products (Figure 6). The direct coupling product between mercaptosilane and the alkene was formed with a lower yield in comparison with that of the reaction between mercaptosilane and a vinyl-alkene. It clearly indicates that also the cis double bond in SSBR can couple to sulfur via a direct mechanism.

The comparison with the observed NR which consists nearly 100% of cis double bonds needs to consider one big difference. The cis double bond of NR has additionally a methyl group as a side group along the polymer chain. This methyl group adjacent to the double-bonded carbon atom in NR has an electropositive effect which enhances the activity of the unsaturated double bonds in the backbone of the polymer chains towards ionic reactions. A radical coupling might be hindered by this electron positive effect. Additionally, a steric hindrance might affect the reaction. Nevertheless, a small amount of a direct coupling to the cis double bond might still occur, supporting the formation of the observed maximum in the cure curve (Figure 11).

**Figure 14:**
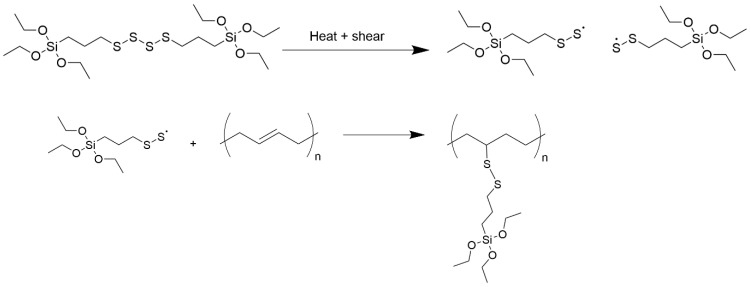
Coupling of TESPT via a sulfur radical to a double bond in a polymeric chain (according to [20]).

Another hint can be found in a study from Holstege [20]. Here, the following mechanism for the reaction of TESPT with a double bond was proposed (Figure 14). It shows that TESPT is first cleaved into fragments carrying a sulfur radical. These radicals can further couple chemically to the double bond of a polymeric chain.

Both examples from the literature show that a direct addition reaction from a sulfur radical (formed from a silane) is possible in a cis double bond in the main chain of an SSBR. For this reason, it can again be considered that the direct sulfur coupling in the case of the curing reaction of BR with sulfur is possible. As Sato [12] already reported, this shows that the reaction of a cis-alkene with a sulfur radical imparts a lower yield than the vinyl-alkene. This can explain the observed behavior in Figure 13: the high-cis BR compound without activators shows a significant increase but still has a lower final torque than the one with activators, meaning that a direct coupling via an S-radical is also possible to cis double bonds but with lower yields.

**Figure 15:**
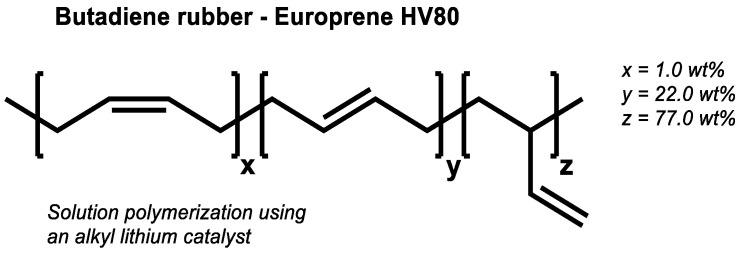
Structure of the used high-vinyl BR type (according to [21,22]).

**Figure 16:**
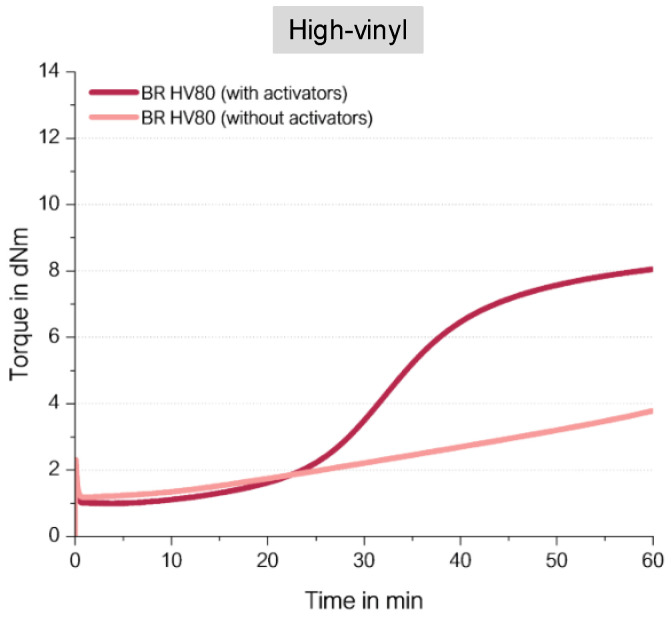
Curing behavior of high-vinyl BR.

It was now expected that the BR with a high-vinyl content (Figure 15) would also show, in the absence of activators, the same final torques that were observed before in the high as well as the low-vinyl SSBR. Surprisingly, a completely different behavior was observed (Figure 16).

The high-vinyl BR compound without activators ends up in a very low final torque. How can this unexpected behavior be explained?

**Figure 17:**
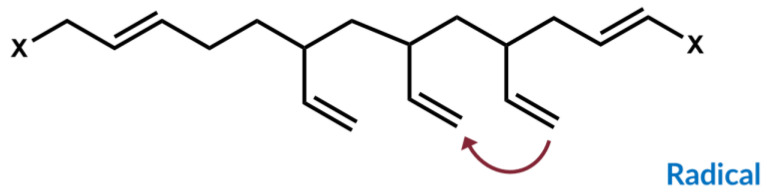
Radical coupling of adjacent vinyl groups.

Radicals are very reactive; they couple to the closest reaction partner. This means that because they have a polymeric structure with adjacent vinyl groups, they react preferably with each other (Figure 17). The presence of such adjacent vinyl groups with a vinyl content of 77% is highly likely. Such an internal crosslink reaction is already known from curing reactions where triallyl cyanurate (TAC) or triallyl isocyanurate (TAIC) are used as co-agents [23]. This means the assumed direct sulfur coupling can take place, but the formation of a radical along the polymeric chain triggers a further coupling reaction to the neighbored vinyl group. In the end, no crosslinks between different polymer chains are formed, which leads to the observed but not pronounced increasing torque.

This leads to the conclusion that an effective direct sulfur coupling needs a “spacer” such as the styrene group in SSBR or a cis or trans BR unit in the low-vinyl BR to hinder such a coupling reaction to adjacent vinyl groups.

By comparing the achieved final torque in Figure 13 and Figure 16 for the BR-compounds with activators, another conclusion can be drawn. The high-vinyl BR shows that even with activators, there is an almost 30% lower final torque than the high-cis BR with activators. This indicates that even in the high-vinyl BR with activators, such an adjacent crosslink reaction might take place, which is ineffective for the increase in crosslink density and therefore also ineffective in causing an increase in torque. This explanation is also true for the observed lower torque for the high-vinyl SSBR in comparison to the low-vinyl SSBR (Figure 9). Additionally, high-vinyl BR or SSBR have much less allylic hydrogen atoms where the “traditional” sulfur coupling reaction via the activated Zn complex can take place [24]. For this reason, the addition reaction towards the double bond is much more likely.

## 8. Curing Behavior of High-Vinyl SSBR/High-Cis BR Filled with Silica/Silane Versus Filled with Carbon Black

**Figure 18:**
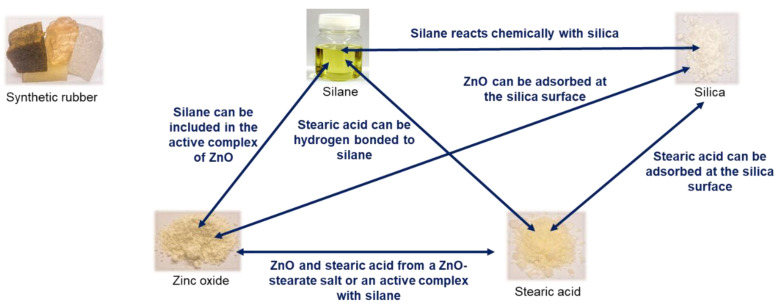
Manifold interactions between ZnO, stearic acid and silica, as well as silane (according to [25]).

Up to now, all investigated compounds did not include any filler. In a silica/silane-filled compound, many interactions are reported between the activators and silica as well as silane (Figure 18) [25].

Furthermore, the coupling of the silane-modified silica to the polymer was also reported to follow the “traditional” sulfur coupling at the allylic position (Figure 4). This coupling was assumed to be supported from the active Zinc complex. As discussed before, this support was considered to be unlikely due to the formation of the Zinc complex at the surface of the non-mobile Zinc oxide.

**Figure 19:**
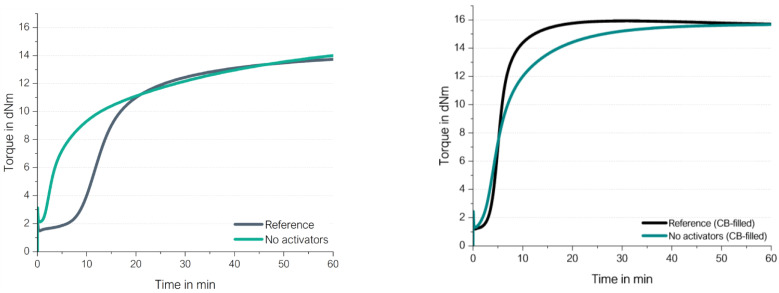
Curing behavior of the silica/silane (**left**) as well as the CB-filled (**right**) high-vinyl SSBR/high-cis BR compounds; “reference” is referring to the compounds with activators.

To find out which influence the silica/silane system has on the direct coupling reaction between a sulfur radical and a vinyl group, a rubber compound with 80 phr high-vinyl SSBR and 20 phr high-cis BR filled with silica/silane (Bis(triethoxysilylpropyl)disulfide (TESPD) was investigated. Two compounds were evaluated: one with curing activators ZnO and stearic acid, and one without these activators. Furthermore, these rubber compounds were based on a model green tire tread compound with 1.5 phr DPG, 2.0 phr TBBS and 1.4 phr sulfur. The compounds were compared with carbon black (CB)-filled high-vinyl SSBR/high-cis BR compounds where such manifold interactions between the different ingredients as described in Figure 18 were not expected. Figure 19 shows the curing behavior of the silica/silane on the left side as well as that of the CB-filled compounds on the right side. The used materials and methods are described in more detail in Appendix A.

The curing of high-vinyl SSBR/high-cis BR without activators (ZnO/stearic acid) also starts in CB-filled SSBR/BR slightly earlier and leads to the same final torque. This means that the direct coupling of the sulfur radical to the vinyl group also does occur in a CB-filled system and the presence of CB influences the curing behavior only slightly.

The silica/silane-filled compounds show the same final torque, independent of whether the activators are present or not. If the activators are left out, the scorch time is significantly reduced. This reduction is more significant in the silica/silane-filled system. This can be explained by the fact that the silica/silane system can absorb and, in this way, deactivate part of the curing system. In the CB-filled compounds, the scorch time is much shorter in both compounds because this filler does not absorb curing chemicals. Both cure curves of the filled compounds without activators look very similar, showing again that the radical reaction starts very fast. We can conclude that the direct sulfur coupling towards a vinyl group also takes place in filled compounds.

## 9. Curing Behavior of High-Vinyl SSBR/High-Cis BR Without DPG

It was shown in the previous described studies that a direct coupling of a sulfur radical towards a vinyl group can take place, even in the absence of ZnO as well as stearic acid. If the assumed direct coupling mechanism shown in Figure 8 on the right side is correct, it can be considered that also DPG can be left out.

**Figure 20:**
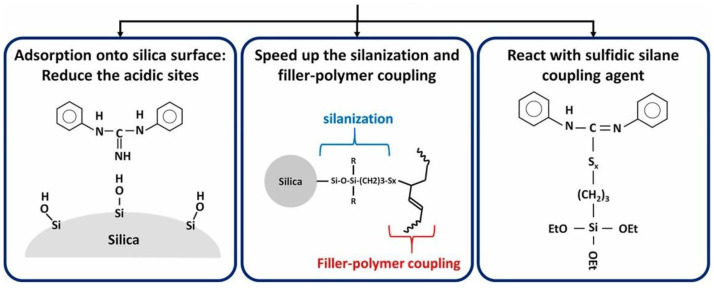
Manifold interactions for DPG in a silica/silane-filled tire tread compound (according to [26]).

DPG plays a manifold role inside a silica/silane compound (Figure 20) [26]. It adsorbs at the silica surface and blocks it for the adsorption of the primary accelerators, and it speeds up the silanization reaction and it can react directly with the silane.

DPG has, for quite some time, been under discussion to be banned from use due to the release of harmful aniline [27]; therefore, it is desirable to find a curing method without DPG, especially in silica/silane compounds.

**Figure 21:**
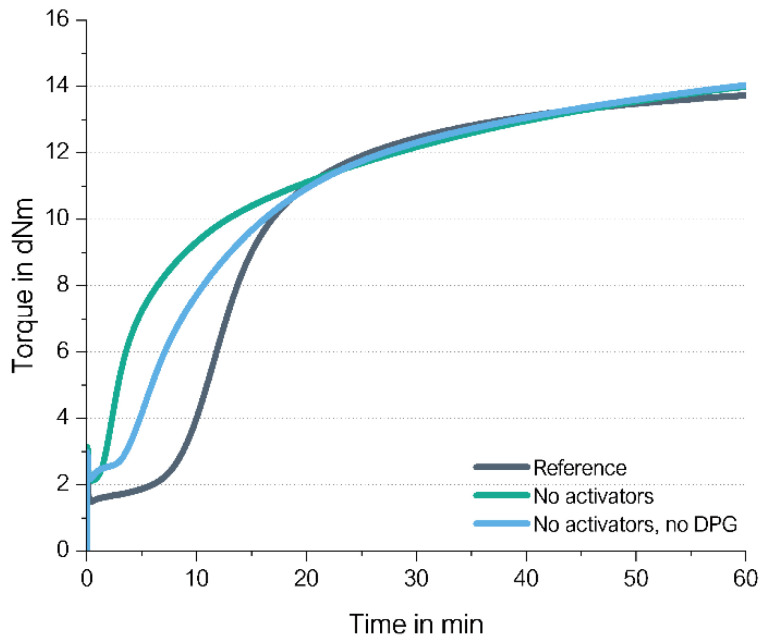
Curing behavior of a silica/silane-filled high-vinyl SSBR with and without activators as well as DPG.

In order to prove if the curing of 80 phr high-vinyl SSBR and 20 phr high-cis BR compounds can occur in the absence of DPG, a silica-filled high-vinyl SSBR-based model green tire tread compound without activators and without DPG was investigated and compared with the same compound but without activators. As a reference, the compound with DPG with or without activators was studied (Figure 21). A model green tire tread compound with 2.0 phr TBBS and 1.4 phr sulfur was selected as the basis for this study.

The curing of high-vinyl SSBR/high-cis BR with activators (ZnO/stearic acid) as well as without activators and DPG starts earlier and leads to the same (slightly higher) final torque. When DPG is left out in addition to the activators, the scorch time is slightly increased, but is still much shorter than the reference. Amines are known for their accelerating effect on the breakage of the sulfur ring, with TBBS having a higher potential than DPG [28]. When DPG is left out, the more active TBBS, or the reaction product from TBBS, the amine, is assumed to be partly adsorbed at the silica surface, being in this way partly immobilized to support the opening of the sulfur ring. The result is the observed longer scorch time.

This means that in a high-vinyl compound not only the accelerators but also DPG can be left out to reach a sufficient crosslink density.

## 10. Curing Behavior of High-Vinyl SSBR Without Any Accelerator

After finding that an effective curing method for high-vinyl SSBR/high-cis BR compounds with no activators as well as with no DPG is required, the following open research question should be answered. Is any accelerator needed for the direct sulfur curing or is the presence of sulfur alone sufficient? In this case, the sulfur radical would be formed only from the sufficient high energy input due to the increase in temperature causing the opening of the sulfur ring.

**Figure 22:**
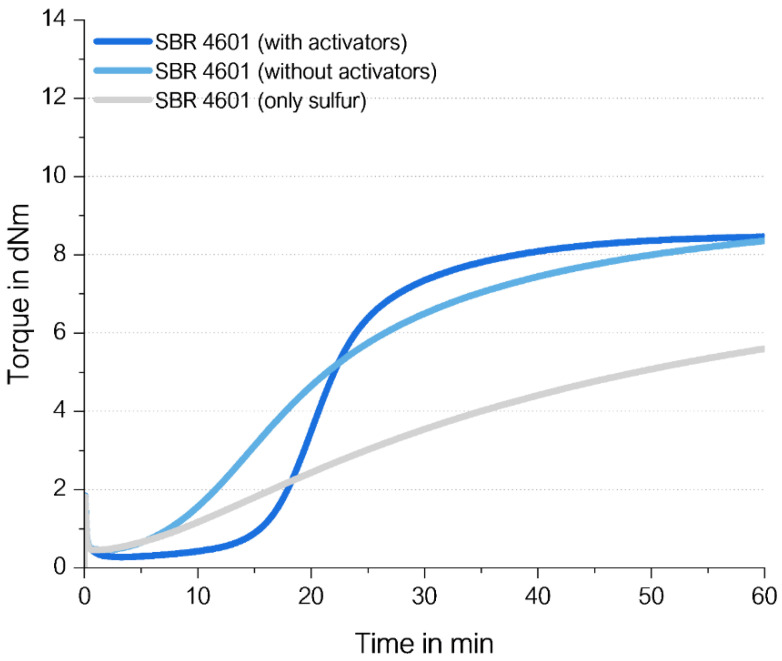
Curing behavior of a high-vinyl SSBR with and without activators as well as without activators and accelerators (named “only sulfur”).

For this, an unfilled high-vinyl SSBR with 1.4 phr sulfur without activators and without any accelerator was investigated and compared to a compound with 2.5 phr ZnO, 2.5 phr stearic acid, and 2.0 phr TBBS (Figure 22). Additional details about the materials and methods are displayed in Appendix A.

The direct sulfur coupling in a compound without activators and without accelerators (gray curve in Figure 22) is slow but leads to 60% of the final torque containing the traditional curing system after 60 min. This means that TBBS seems to support the formation of an S-radical but a direct coupling also takes place in the absence of any other ingredients; however, the formation of sulfur radicals using only heat takes longer.

## 11. Reinforcing Behavior of Filled High-Vinyl SSBR/High-Cis BR Compounds Without Activators

**Figure 23:**
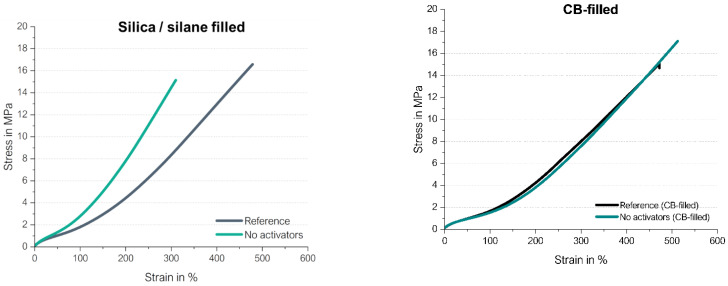
Stress–strain behavior of silica/silane (**left**) as well as CB-filled (**right**) compounds with and without activators.

Up to now, only the cure behavior has been studied in detail. To obtain the final proof that the observed increase in the final torque is indeed related to a good reinforcing behavior, the stress–strain curves of the before described silica/silane filled as well as carbon black-filled compounds were measured (Figure 23). They were based on a model green tire tread compound with 1.5 phr DPG, 2.0 phr TBBS and 1.4 phr sulfur and compared with the same compound where additional activators were added (called “reference” compound). Appendix A includes additional information about the used materials and methods.

The carbon black-filled compounds show that either with and without activators, there is the same reinforcing behavior, and only the elongation at break as well as the tensile strength are higher for the compounds without activators. In the case of the silica/silane-filled SSBR/BR, a higher modulus but shorter elongation at break as well as tensile strength are achieved in the absence of activators. This shows that the sulfur is more effectively used for crosslinks in the absence of activators. If ZnO and stearic acid are present, manifold interactions can take place between the filler system, also covering unwanted reactions like the hydrogen bonding between silane and stearic acid. The sulfur curing can take place via the direct coupling as well as the “traditional” ionic pathway, leading to lower crosslink densities.

This assumption was confirmed from swelling tests: The silica-filled compound without activators results in a higher crosslink density than one with activators. For the shown example in Figure 23, this is a difference in the crosslink density of 119 mol/cm³ for the reference compound with activators versus that of the compound without activators of 166 mol/cm³. For the CB-filled compounds, the reference shows a crosslink density (CLD) of 131 mol/cm^3^, while the compound without activators resulted in a CLD of 122 mol/cm^3^. The more similar stress–strain behavior for the CB-filled compounds can therefore be explained by the more similar CLD values. The results in the CB-filled compound are more comparable to each other because the CB does not absorb other rubber ingredients. In addition, no coupling agent like silane is included in the CB-filled compound, resulting in fewer interactions between the rubber ingredients and the activators.

All in all, cured high-vinyl SSBR/high-cis BR compounds without activators show a high reinforcing behavior, similar to the reference when filled with carbon black, with increased moduli when filled with silica/silane.

## 12. Summary

NR is able to couple via the well-investigated activated complex where ZnO and stearic acid as well as accelerators and sulfur are involved.

**Figure 24:**
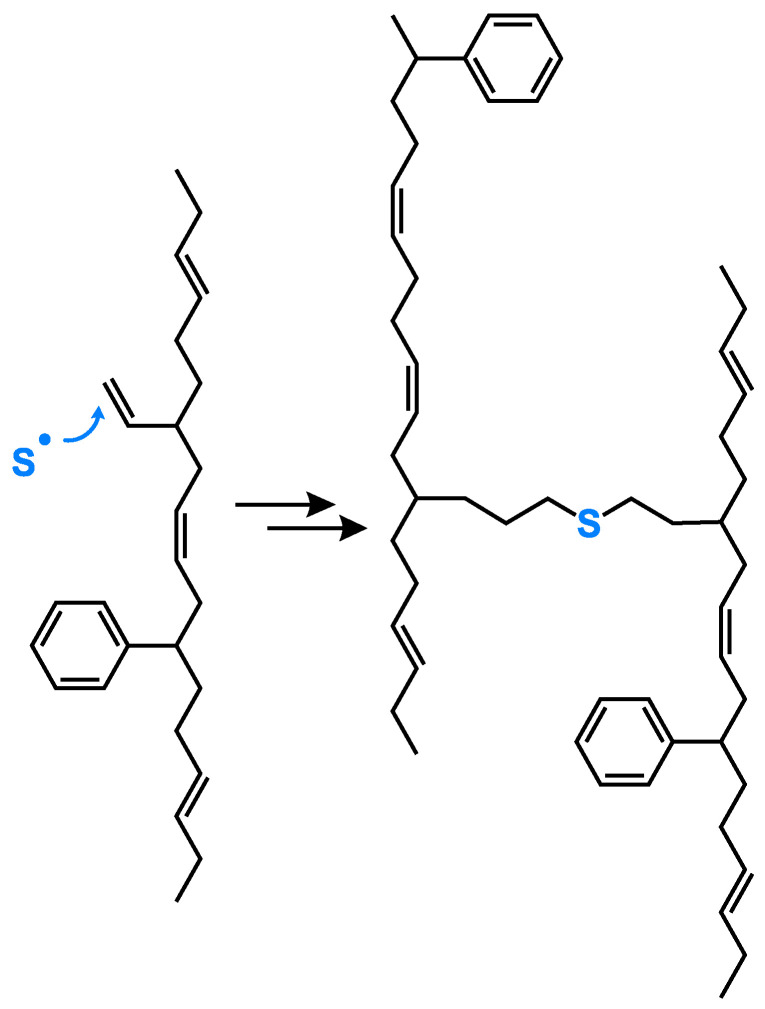
Direct sulfur coupling mechanism in SSBR.

A different sulfur coupling mechanism occurs in high-vinyl SSBR than in NR (Figure 24).

The sulfur couples by a high amount to vinyl via a sulfur radical. The coupling to a cis double bond in SBR can occur as well via a direct sulfur coupling but in a lower amount. For this coupling reaction, no activators (ZnO/stearic acid) are required. Their absence leads to a shorter scorch time. This means that ZnO and stearic acid act as retarders, not as activators in this system like in a traditional coupling system. The scorch time should be adjusted for practical use, but it is already sufficiently long without both activators and DPG.

As an overall conclusion, a silica/silane filled tire tread compound based on high-vinyl SSBR/high-cis BR shows the same curing performance even when the typically used activators as well as DPG are left out. Only adding one primary accelerator like TBBS plus sulfur is required for the whole curing system of the compound.

The absence of activators leads to similar (CB-filled) yet higher (silica/silane-filled) reinforcement in a model tire tread compound.

## 13. Conclusions

Until now, it was assumed that the sulfur coupling in a rubber compound followed a mechanism where the coupling to the polymer chain occurs in the allylic position, supported by a Zinc complex. This study showed that a direct coupling via a sulfur radical is the dominant mechanism in a high-vinyl SSBR compound, the typical main elastomer for a tire tread compound. The generally assumed coupling to the allylic position is only valid, e.g., in NR, when there are no vinyl groups.

The direct sulfur coupling in a high-vinyl SSBR does not need any activators nor DPG; the presence of an activator for the sulfur ring opening like TBBS is sufficient. It can be considered that the coupling of the silane to the polymer, attached already to the silica surface, follows the same mechanism, a direct coupling via a sulfur radical.

Therefore, a new tire tread compound should be based on high-vinyl SSBR/high-cis BR without ZnO, without stearic acid and without DPG. With this, the manifold interactions in the typical silica/silane filled green tire tread compound are significantly reduced, avoiding unwanted reactions like the interaction of stearic acid with silane which negatively influences the silica/silane coupling.

The abraded wear particles from such new tires will be less harmful for the environment. These tire wear particles do not contain any Zn species which are toxic to aquatic systems. The use of DPG can also be avoided, since this releases harmful aniline. This will have a very positive effect on the overall microplastic pollution caused by tire wear.

Furthermore, the big particles of ZnO are not well dispersed in the rubber matrix. By leaving them out, the compounds do not contain these inhomogeneous spots which can be the origin of abrasion. For this reason, the wear resistance from such tire tread compounds is expected to be better.

**Figure 25:**
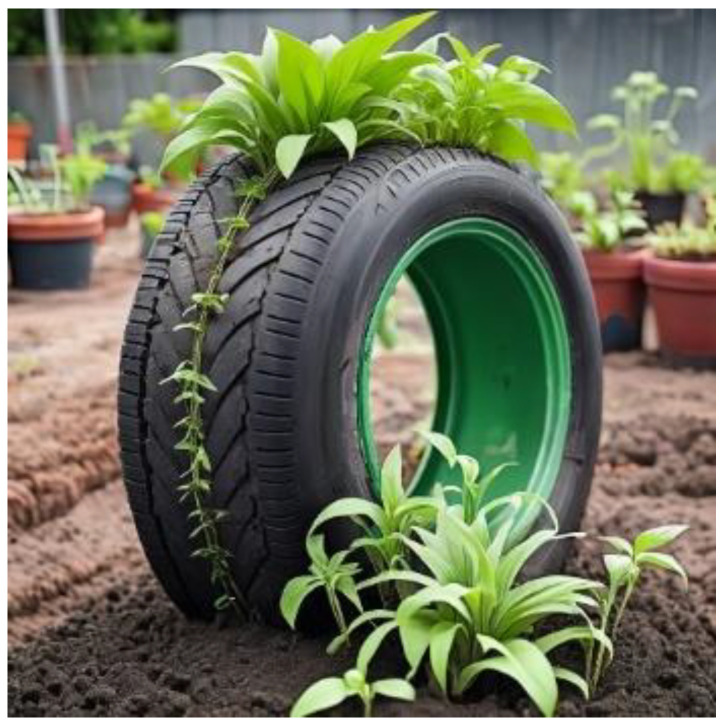
Sustainable and plant-based tire [A. Blume created with AI].

This new tire tread compound contains less ingredients which also reduces the production costs. It leaves two environmentally critical substances out: Zinc species as well as DPG. This unique new approach of reducing the number of substances without compromising the in-rubber performance enables the design of a more sustainable, more eco-friendly tire tread compound and paves the way for further innovations (Figure 25).

## Figures and Tables

**Table 1:** Selected SSBR types.

	SSBR Sprintan SLR 3402	SSBR Sprintan SLR 4601
Styrene content in %	15	21
Vinyl content in %	30	63
Functionalization	yes	yes

## Data Availability

Data are contained within the article.

## Appendix A

Materials:

Five different polymers were used in this study: SSBR SPRINTAN^®^ SLR 4601 and SSBR SPRINTAN^®^ SLR 3402 (Synthos Schkopau GMBH, Schkopau, Germany), BR Buna^®^ CB24 (Arlanxeo, Dormagen, Germany), BR EUROPRENE^®^ HV80 (Versalis S.p.A., San Donato Milanese, Italy) and NR SMR10 (WEBER & SCHAER GmbH & Co. KG, Hamburg, Germany). Table A1 displays additional information about these polymers.

**Table A1:** Additional data on the used polymers.

Polymer Type	Cis 1,4 Content	Vinyl 1,2 Content	Styrene Content
SLR 4601	x	62%	21%
SLR 3402	x	30%	15%
CB24	>96%	x	x
HV80	x	77%	x
SMR10	>99%	x	x

Data obtained from technical data sheets provided by the suppliers.

For fillers, ULTRASIL^®^ 7000GR (Evonik Industries, Wesseling, Germany) and carbon black CORAX^®^ N220 (Orion Engineered Carbons GmbH, Cologne, Germany) were used. Bis(triethoxysilylpropyl) disulfide (Evonik Industries, Wesseling, Germany) was selected as the silane and Treated Distillate Aromatic Extract (Hansen & Rosenthal, Hamburg, Germany) as the plasticizer. The curing package consisted of Zinc oxide (Umicore Zinc Chemicals, Angleur, Belgium), stearic acid (Emery Oleochemicals GmbH, Düsseldorf, Germany), sulfur (Zolfindustria, Trecate, Italy), *N*-tert-butyl-benzothiazole sulfonamide (General Química S.A., Alva, España) and *N*,*N*’-diphenylguanidine (MLPC International (Arkema Group), Rion-des-Landes, France).

Mixing procedure:

The compounds were mixed according to the mixing procedure as shown in Table A2. The compounds were mixed using a Brabender Plasticorder 350S (Brabender GmbH & Co. KG, Duisburg, Germany). After each mixing stage, the rubber compounds were sheeted out on a two roll mill.

**Table A2:** Mixing procedure.

Time in m:s	Action
Stage 1: pre-heating 80 °C, 70 rpm, fill factor 72%	
0:00	Addition of rubber
1:00	Addition of 2/3 silica, 2/3 silane
2:30	Addition of 1/3 silica, 1/3 silane, ZnO, stearic acid, and plasticizer
4:00	15 s sweep
4:15	Increase in torque (increase temperature to 130 °C)
7:00	Stop mixing (reaching 140 °C)
Stage 2: pre-heating 80 °C, 80 rpm, fill factor 69%	
0:00	Addition of elastomer masterbatch
0:50	Addition of DPG
1:00	Increase in torque (increase temperature to 130 °C)
5:00	Stop mixing (reaching 140 °C)
Stage 3: pre-heating 50 °C, 50 rpm, fill factor 66%	
0:00	Addition of elastomer masterbatch, curatives (sulfur and TBBS)
3:00	Stop mixing

Rheometer curves:

The Rubber Process Analyzer RPA-Elite (TA Instruments, New Castle, DE, USA) was used to measure the rheometer curves as displayed in this article. The measurement was conducted at a frequency of 1.667 Hz, a deformation of 6.98%, and a temperature of 160 °C. The curing behavior was monitored for one hour.

Additional methods used for filled compounds:

The stress–strain behaviors of the silica/silane and carbon black-filled compounds were measured according to ISO 37 [29] using dumbbell type 2 shaped specimens, which were cured according to the t90. Additionally, the crosslink density of the compounds was calculated. For these five cured samples, ~0.3 g of each compound were prepared. These samples were swollen for 7 days in 150 mL of toluene at room temperature. Consequently, the crosslink density was calculated with the Flory–Rehner Equation (A1) [30]. The average value of the five samples was determined and evaluated in this study.

(A1) $$v=-\frac{\ln\left(1-V_{r\;}\right)+V_{r\;}+\chi V_{r}^{2}}{V_{0}\left({V_{r}}^{\frac{1}{3}}-\frac{{2V}_{r}}{f}\right)}$$

where

Vr = volume percentage/fraction of rubber in a swollen sample;

V0 = the molar volume of the solvent used (106.9 cm^3^/mol for toluene);

f = the functionality of crosslinks (assuming that tetra-functional crosslinks are formed, f = 4);

χ = the Flory–Huggins rubber–solvent interaction parameter (for SBR in toluene, χ = 0.378 [31]);

v = the crosslink density per unit volume (mol/cm^3^)
